# Novel Transgenic Mice for Inducible Gene Overexpression in Pancreatic Cells Define Glucocorticoid Receptor-Mediated Regulations of Beta Cells

**DOI:** 10.1371/journal.pone.0030210

**Published:** 2012-02-17

**Authors:** Bertrand Blondeau, Iman Sahly, Emmanuelle Massouridès, Amrit Singh-Estivalet, Bérengère Valtat, Delphine Dorchene, Frédéric Jaisser, Bernadette Bréant, Francois Tronche

**Affiliations:** 1 INSERM UMR-S 872, Centre de Recherches des Cordeliers, Paris, France; 2 Université Pierre et Marie Curie, Paris, France; 3 Centre National de la Recherche Scientifique, UMR7224, “Molecular Genetics, Neurophysiology and Behavior” team, Paris, France; 4 Institut National de la Santé et de la Recherche Médicale, U952, Paris, France; University of Bremen, Germany

## Abstract

Conditional gene deletion in specific cell populations has helped the understanding of pancreas development. Using this approach, we have shown that deleting the glucocorticoid receptor (GR) gene in pancreatic precursor cells leads to a doubled beta-cell mass. Here, we provide genetic tools that permit a temporally and spatially controlled expression of target genes in pancreatic cells using the Tetracycline inducible system. To efficiently target the Tetracycline transactivator (tTA) in specific cell populations, we generated Bacterial Artificial Chromosomes (BAC) transgenic mice expressing the improved Tetracycline transactivator (itTA) either in pancreatic progenitor cells expressing the transcription factor *Pdx1* (BAC-Pdx1-itTA), or in beta cells expressing the *insulin1* gene (BAC-Ins1-itTA). In the two transgenic models, itTA-mediated activation of reporter genes was efficient and subject to regulation by Doxycycline (Dox). The analysis of a tetracycline-regulated LacZ reporter gene shows that in BAC-Pdx1-itTA mice, itTA is expressed from embryonic (E) day 11.5 in all pancreatic precursor cells. In the adult pancreas, itTA is active in mature beta, delta cells and in few acinar cells. In BAC-Ins1-itTA mice tTA is active from E13.5 and is restricted to beta cells in fetal and adult pancreas. In both lines, tTA activity was suppressed by Dox treatment and re-induced after Dox removal. Using these transgenic lines, we overexpressed the GR in selective pancreatic cell populations and found that overexpression in precursor cells altered adult beta-cell fraction but not glucose tolerance. In contrast, GR overexpression in mature beta cells did not alter beta-cell fraction but impaired glucose tolerance with insufficient insulin secretion. In conclusion, these new itTA mouse models will allow fine-tuning of gene expression to investigate gene function in pancreatic biology and help us understand how glucocorticoid signaling affects on the long-term distinct aspects of beta-cell biology.

## Introduction

The pancreas is a complex organ composed of both exocrine and endocrine cells. The exocrine cells make up the majority of the pancreas and are grouped together into a highly branched ductal system that produce and secrete enzymes into the digestive tract. The endocrine portion is composed of four major cell types, alpha, beta, delta and pancreatic polypeptide (PP) cells that secrete into the bloodstream glucagon, insulin, somatostatin and PP respectively. The endocrine cells that account for approximately 1% of the volume of the pancreas are grouped as clusters of cells named islets of Langerhans, which are dispersed in the exocrine tissue (for review see [1]).

All pancreatic cells arise from common precursors present at an early stage of embryonic development. The differentiation of these precursors into mature cells requires controlled expression of transcription factors as well as factors produced both by the pancreas and the surrounding tissues [2].

In recent years, great knowledge about genes that regulate pancreatic development and beta-cell function has been gathered, mostly thanks to the use of transgenic mice allowing Cre-mediated DNA recombination in specific pancreatic cell populations. Using this approach, we have shown that deletion of the glucocorticoid receptor (GR) gene in beta cells (using RipCre mice) had no effect on beta-cell mass whereas GR gene deletion in pancreatic precursors (using PdxCre mice) led to a doubled beta-cell mass [3]. These results, together with previous findings in rats [4], clearly defined glucocorticoids as major inhibitors of beta-cell development. More recently, we provided genetic evidence that the GR was required for the deleterious effects of fetal undernutrition on beta-cell development [5]. Altogether, these results highlight the importance of generating and using mouse models that allow for gene modifications not only in specific cell populations but also during specific time frames.

The tetracycline-dependent regulatory (*tet*) system allows a precise spatial and temporal control of transgene expression [6]. In the present study, we applied this approach to pancreatic cells. The *tet* system relies on two components: a transgene that allows the expression of the tetracycline-controlled transactivator (tTA) and a transgene that contains a gene of interest under the control of a synthetic tTA-dependent promoter (*tetO*). In the absence of tetracycline or analog molecules (such as Doxycycline or Dox), tTA binds to the *tetO* DNA sequences, allowing activation of the tTA-dependent promoter and transcription of the gene of interest. In the presence of Dox, tTA undergoes a conformational switch and cannot interact with its DNA target and transcription of the gene of interest does not occur. This system provides several advantages: it combines the possibility of expressing a gene in a cell-specific manner and stopping its expression by Dox administration.

We generated two novel transgenic mouse lines expressing an improved version of the tTA, the itTA gene [7]. Since the most efficient approach to express a transgene is to use large DNA segments, we placed the itTA coding sequence under the control of the regulatory elements of either the *Pdx1* or the *Insulin1* genes using bacterial artificial chromosomes (BAC) spanning the entire genomic regions of *Pdx1* or *Insulin1* genes. We show that in these mice, itTA mediates the activation of a reporter gene specifically in *Pdx1-* or *Insulin1-*expressing cells. We also show that its activity can be regulated by Dox administration thus allowing the reversible expression of targeted genes. We then examined the consequences of conditional GR overexpression in a specific pancreatic cell populations. Our results demonstrate that these mouse lines will be exquisite tools for the development of mouse models to study how specific genes modulate pancreas development and function.

## Materials and Methods

### Mouse husbandry

Animals were bred and raised under standard animal housing conditions, in a 12-h light/dark cycle (7am–7pm), temperature (22±1°C) and humidity (60±5%). Food and water were available *ad libitum*. All experiments were performed in accordance with French (Ministère de l'Agriculture et de la Forêt, 87-848) and European Economic Community (EEC, 86-6091) guidelines for care of laboratory animals. The experiments were examined and approved by the Regional Ethics Committee in Animal Experiment N°3 of Ile-de-France region (reference p3/2008/012).

### Generation of transgenic mice

#### Pdx1-itTA Mice

Using the Ensembl genome database, we chose a 196 kb mouse BAC RP24-367D23 containing the *Pdx1* gene from C57BL/6J BAC library. The corresponding clone was obtained from CHORI-USA (http://www.chori.org) and was modified by subsequent homologous recombination steps in EL250 bacteria according to Lee et al. [8]. This clone contains two other genes, *Cdx2* and *Flt3*. In order to avoid any gene dosage effects due to the presence of extra copies, these two genes in the transgenic mice were inactivated. The *Cdx2* first exon including the ATG initiation site was initially replaced by an *ampicillin* resistance gene flanked with two modified FRT (Flippase Recognition Target) DNA sequences (FRT3) that cannot recombine with classical FRT sites [9]. The homologous recombination cassette was amplified by PCR from the plasmid “pIntron-polyA FRT3-NotI-Amp- FRT3” (kindly provided by Dr. E. Casanova) using the following primers: *Cdx2* 5′ homology arm:

5′*AGCTACCTTCTGGACAAGGACGTGAGCATTTATCCTAGCTCCGTGCGCCA*AAGAACCAGCTGGGGCTCGA3′, *Cdx2* 3′ homology arm:

5′*CTTCCTAGGGACTGCTGCGCGGGCTTCCGAATCCACTCGCACAGGTTTCG*GAATGCAGCTAGCCGTTAAT 3′. Each primer consists of 20 base homologues to the *ampicillin* DNA sequence and 50 bases corresponding to the *Cdx2* gene homology arms (in italics). The amplified targeting cassette thus contained the *ampicillin* gene coding sequence flanked by two FRT3 and two *Cdx2* gene homology arms located upstream and downstream of the ampicillin-FRT3 DNA sequence.

The recombination was performed as previously described [8]. Briefly, EL250 bacteria were transformed with 500 ng BAC DNA by electroporation. Subsequently, the BAC-containing EL250 bacteria were incubated 15 min at 42°C to induce recombination enzymes and transformed with 400 ng of the targeting cassette. Transformants were selected on ampicillin (100 µg/ml) and chloramphenicol (12.5 µg/ml) plates. Correctly targeted colonies were identified by restriction site analysis. Flippase expression from EL250 cells was then induced by incubating the cultures with 0.1% L-arabinose for 1 h allowing the excision of the *ampicillin* gene. The bacteria were plated on ampicillin to test for the loss of ampicillin resistance. The BAC was purified an analyzed for adequate modification. The resulting BAC was submitted to a second homologous recombination step to introduce the *itTA* coding sequence (gift of Rolf Sprengel, MPI Heidelberg) into the BAC at the *Pdx1* ATG initiation site and until 188 nucleotides (nt) after thus disrupting the *Pdx1* Open Reading Frame (ORF). To create the targeting cassette, *Pdx1* homology arms were generated and subcloned into a pRK5-itTA plasmid containing the *itTA* coding sequence, and a polyA transcriptional termination signals, followed by the *ampicillin* gene flanked by two FRT sites in the same orientation. The 5′ homology sequences of 200-bp was amplified by PCR (primer-F 5′GCGGGGTACCGGCCAATGATGGCTCCAGGG, primer-R: 5′-AACCATCGATTGGGGGCCAGCAGCCCCGGG), digested with Kpn1 and Cla1 and cloned into the corresponding sites upstream of the ATG codon of the *itTA* gene. Similarly, a 200-bp DNA segment located within the *Pdx1* ORF was amplified by PCR (primer-F: 5′ATAGGCTAGC CATCTCCCCATACGAAGTGC, primer-R: 5′- GCATGTATACTGGTGGATTTCAGCCACGGG), digested with Nhe1 and BstZ1, and subcloned downstream of the *ampicillin* sequence. The targeting cassette was introduced into the BAC by homologous recombination as described above. Correctly recombined BAC clones were identified by DNA restriction analysis and pulsed-field gel electrophoresis.

After removal of the ampicillin selection cassette, BAC DNA was digested with BsiW1 resulting in the inactivation of *Flt3* gene and excision of a 165 kb long DNA fragment. This fragment was then purified on Sepharose CL4b (Pharmacia) chromatography columns and used for pronuclear injections into C57BL/6J zygotes performed at the Service des Animaux Transgéniques (SEAT, Villejuif, France) of the Centre National de la Recherche Scientifique.

The BAC-Pdx1-itTA transgenic line has been deposited at the European Mouse Mutant Archives (http://www.emmanet.org/).

#### Ins1-itTA mice

A mouse BAC RP23-401C13 clone containing the *Insulin1* gene from C57BL/6J BAC library was chosen using Ensembl genome database and obtained from CHORI-USA. The BAC clone was modified as described above to introduce at the ATG of the gene a cassette positioning the ATG of the *itTA* gene followed by an *ampicillin* resistance gene, flanked by two FRT sites. To create the targeting cassette, *Insulin1* 5′ and 3′ homology arms were generated and subcloned into pRK5-itTA plasmid containing *itTA* coding sequence followed by the *ampicillin* gene flanked by two FRT sites in the same orientation. The polyA-transcriptional termination signal sequence initially present in the plasmid was removed by BamH1 and Xho1 digestion. The *Insulin1* 5′ homology sequence of 200-bp was amplified by PCR (primer-F: 5′CGGGGGTACCACTATAAAGCTGGTGGGCAT, primer-R: 5′GCCCATCGATGAAAGATAGGCAGGGTTGAG), digested with Kpn1 and Cla1 and cloned into the corresponding sites upstream of the ATG codon of the *itTA* gene. Similarly, a 200-bp DNA segment located within the *Insulin1* ORF was amplified by PCR (primer-F: 5′ATAGGCTAGCGCTTCTTCTACACACCCAA, primer-R: 5′GACTGTATACTAGTTCTCCAGCTGGTAGAG), digested with Nhe1 and BstZ1 and subcloned downstream of the *ampicillin* sequence. The targeting cassette was excised by Kpn1 and BstZ1 digestion, and inserted by homologous recombination into the BAC, replacing a fragment beginning 3 nucleotides before the *Insulin1* ATG and ending 314 nt after. This resulted in the substitution of the *Insulin1* gene ORF by the *itTA* gene. The restriction pattern of the BAC DNA was checked by pulsed-field gel electrophoresis before and after each modification step. The 190-kb insert was excised by Not1 digestion and purified on Sepharose CL4b (Pharmacia) chromatography columns before injection into DBA/FVBN zygotes performed at the Service des Animaux Transgéniques (SEAT, Villejuif, France) of the Centre National de la Recherche Scientifique.

The BAC-Ins1itTA transgenic line has been deposited in the European Mouse Mutant Archives

### Generation of double transgenic mice

Tg(BAC-Pdx1-itTA);Tg(LacZtetOhGR) and Tg(BAC-Ins1-itTA);Tg(LacZtetOhGR) double transgenic mice were obtained by crossing transgenic mice from the Pdx1-itTA or Ins1-itTA lines with mice bearing a transgene that contains the lacZ and the human GR genes under the control of the tetracycline response element, the LacZtetOhGR mice that were previously described [10]. Transgenic animals were identified by PCR amplification of genomic DNA prepared from tail biopsy using the following primers: For Pdx1-itTA: forward primer GCGGGGTACCGGCCAATGATGGCTCCAGGG and reverse primer GTCCTGCCAGGACTCCCCTTCCAGAGGGCA. For Ins1-itTA: forward primer CGGGGGTACCACTATAAAGCTGGTGGGCAT and same reverse primer than for Pdx1-itTA. For the presence of LacZtetOhGR we used the following primers: forward primer GTCGTTTTACAACGTCGTGACT and reverse primer GATGGGCGCATCGTAACCGTGC.

### Analysis of LacZ expression

Pancreata were collected from adult male mice. For early embryonic stages (embryonic days E11.5 or E13.5), analysis of beta-galactosidase expression, encoded by the LacZ gene, was performed on digestive blocks. Briefly, fetal tissues were excised, fixed for one hour in 4% paraformaldehyde, and incubated overnight in 40 mg/ml Xgal (Sigma) solution as previously described [11]. For postnatal pancreata, organs were dissected, fixed for 1 h in 4% paraformaldehyde, cryoprotected overnight in 30% sucrose, frozen and 10 µm-frozen sections were prepared. Xgal (40 mg/ml) staining was performed on sections that were subsequently counterstained with 1% eosin solution and mounted with Glycergel (Dako).

### Immunofluorescence

For localization of Xgal staining on adult pancreatic sections, Xgal staining was performed as described above and sections were subsequently incubated overnight with antibodies raised against insulin (Dako), glucagon (Sigma), Somatostatin (Sigma), PP (Sigma) or amylase (Sigma). Sections were then incubated for 1 h with appropriate secondary antibodies coupled to fluorescein or Texas Red (Jackson Immunoresearch). Pictures were taken on a DMRB fluorescent microscope with a DFX350 digital camera (Leica).

### Immunohistochemistry

Staining for the GR was performed on pancreatic sections from Pdx1-itTA/ LacZtetOhGR, Ins1-itTA/LacZtetOhGR and control (LacZtetOhGR) mice with an anti-GR antibody M20 (Santa Cruz Biotechnologies). Secondary antibody coupled to horseradish peroxidase (Jackson Immunoresearch) was used and revealed with DAB+ (Dako).

### Treatment with doxycycline

Control of itTA activity and lacZ expression was achieved by giving Dox to mice in the drinking water at 0.1 mg/ml when given to pregnant females or at 1 mg/ml when given to other adult mice.

### Beta-cell mass and glucose homeostasis in double mutant GR overexpressing mice

These experiments were performed in male adult mice. Beta-cell fraction and mass were assessed as previously described [5]. Briefly, pancreata from male animals were dissected, fixed in formalin, embedded in paraffin and entirely sectioned at a thickness of 5 µm. Eight sections taken at regular intervals throughout the whole pancreas were then stained for insulin. Beta-cell fraction was defined as the ratio of the insulin staining to the whole tissue area, both parameters analyzed on a Leica QWin500 system. Beta-cell mass was obtained by multiplying the beta-cell fraction by the pancreatic weight. To assess glucose homeostasis, intra-peritoneal glucose tolerance tests were performed on overnight-fasted mice by injecting glucose at 2 g/kg body weight and measuring blood glucose levels before and 15, 30, 60 and 120 minutes after glucose injection. Serum insulin concentrations were measured by ELISA (Mercodia).

### Statistical analysis

Results are expressed as means ± SD. The statistical significance of variations was tested by a Mann-Whitney non-parametric test. *p* values<0.05 were considered significant.

## Results

### Generation and characterization of Pdx1-itTA transgenic mice

The tTA system requires two independent lines of transgenic mice: one line expresses the tTA under the control of a specific promoter, and a second line carries a tTA-responsive tetO promoter linked to the target gene of interest. When the two transgenes are present in the same mouse, the tetO-controlled gene is activated only in the cells expressing the tTA. The expression of the target gene can be suppressed by doxycycline.

We used a transgene based on a modified Bacterial Artificial Chromosome (BAC) clones to direct the expression of improved tetracycline-controlled transactivator (itTA) [7], specifically in *Pdx1-* or *Insulin1-* expressing cells. Such a strategy has proved to be more efficient than classical constructs to target a correct expression of the transgene [11], [12], [13].

**Figure 1:**
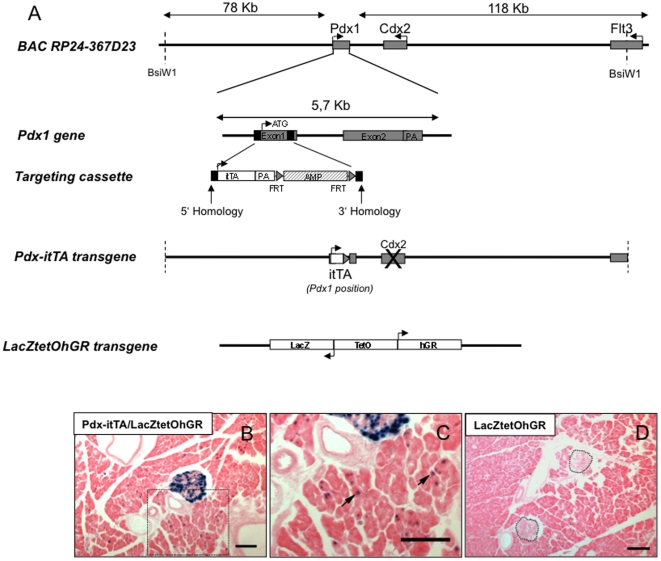
Generation and characterization of mice expressing the itTA under the control of *Pdx1* regulatory elements. (A) Bacterial Artificial Chromosomes (BAC) containing 202 kb of the genomic region of Pdx1 was obtained from a BAC library developed by the CHORI, Oakland, USA. The coding region of the gene was removed by homologous recombination in bacteria and replaced by the itTA cDNA. The construct was then injected in the pronuclei of fertilized eggs. itTA = improved tetracycline transactivator; AMP = ampicillin resistance gene; PA = polyadenylation site; FRT = Flippase recognition target. Below, a scheme representing the LacZtetOhGR construct (B) Lac Z expression revealed by Xgal staining (blue) in islets and in exocrine tissue in Pdx-itTA/LacZtetOhGR mice. (C) Magnified view of the boxed area of B with arrows pointing at blue cells in the exocrine tissue. (D) Absence of blue staining in control mice carrying only the LacZtetOhGR transgene. Two islets are outlined. Scale bar = 50 µm.

Using the Ensembl genome database, we chose two BAC clones (RP24-367D23) and (RP23-401C13) encompassing the entire mouse *Pdx1 or Insulin1* gene respectively. The Pdx1 clone contained 78 kb of DNA sequence upstream from the *Pdx1* gene start codon and 118 kb of DNA downstream of the polyadenylation signals sequence of the gene (Fig. 1A). The clone contains also the *caudal-related homeobox 2* gene (*Cdx2*) and the *FMS-like tyrosine kinase-3* (*Flt3*) gene. To avoid any gene dosage effects and considering that *Cdx2* is expressed in the developing intestine [14] and could eventually interfere with pancreatic development, both genes were inactivated in the BAC. The BAC was further modified to substitute, from the ATG, the *Pdx1* gene with the *itTA* ORF. We chose the *itTA* gene that encodes the same protein than tTA but with an improved codon usage [7]. These transgenic mice were named Tg:BAC-Pdx1-itTA and will be referred in the manuscript as Pdx1-itTA mice.

A target transgene, which carries a bidirectional TetO promoter controlling the simultaneous expression of the LacZ and the human GR (hGR) genes, was previously developed [10] (Fig. 1) and has been used here to characterize itTA activity. Double transgenic mice were obtained by crossing either the Pdx1-itTA or the Ins1-itTA lines with the LacZtetOhGR line carrying the bidirectional tetO promoter [10].

To evaluate the spatial and temporal control conferred by the inducible itTA, beta-galactosidase expression was assayed in adult Pdx1-itTA/LacZtetOhGR mice. We observed a marked expression of beta-galactosidase gene as revealed by Xgal staining in pancreatic islets (Fig. 1B) as well as a sparse staining in pancreatic exocrine cells (Fig. 1C, arrows). No staining was observed in control LacZtetOhGR mice (Fig. 1D) demonstrating that the lacZ expression is dependent upon the presence of the itTA. Analysis of other organs revealed itTA mediated expression of beta-galactosidase in very sparse cells of the stomach, duodenum and kidney (data not shown). This result is in agreement with *Pdx1* expression in few cells of the stomach and duodenum [15]. However, no expression of *Pdx1* has been so far reported in the kidney.

**Figure 2:**
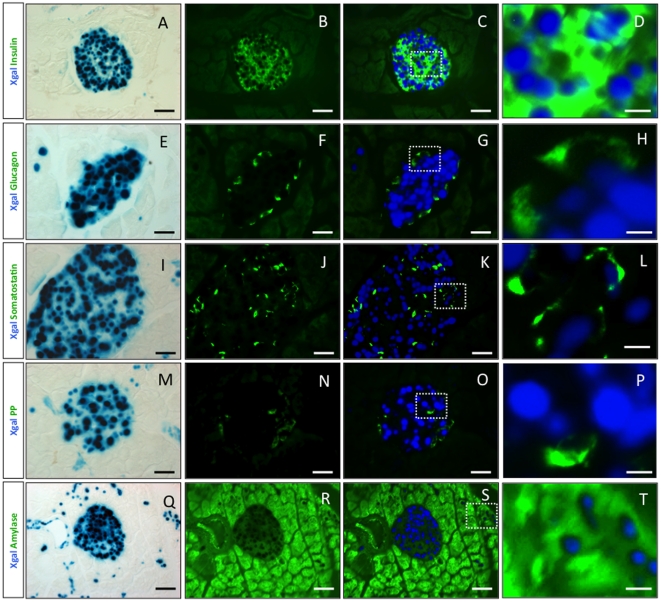
The Pdx1-itTA activates lacZ expression in beta, delta and in acinar cells. (A) (E) (I) (M) and (Q) LacZ expression revealed by Xgal staining (blue) on adult pancreatic sections from a Pdx1-itTA/LacZtetOhGR mouse. (B) Immunofluorescence for insulin (green), (C) merge of A and B and (D) magnified view of inset in C. (F) Immunofluorescence for glucagon (green), (G) merge of E and F and (H) magnified view of inset in G. (J) Immunofluorescence for somatostatin (green), (K) merge of I and J and (L) magnified view of inset in K. (N) Immunofluorescence for PP (green), (O) merge of M and N and (P) magnified view of inset in O. (R) Immunofluorescence for amylase (green), (S) merge of Q and R and (T) magnified view of inset in S. Scale bar = 50 µm except for D, H, L, P and T where scale bar = 10 µm.

To define the nature of beta-galactosidase-expressing cells in the adult pancreas, we performed immunofluorescence on pancreatic sections from adult Pdx1-itTA/LacZtetOhGR mice (Fig. 2). We observed blue staining reflecting the expression of the itTA in all insulin-expressing cells (Fig. 2C and D) and in a fraction of somatostatin-expressing cells (Fig. 2K and L). In contrast, no blue staining was detected in the glucagon- (Fig. 2G and H) or PP- (Fig. 2O and P) expressing cells. Yet, we observed few scattered blue amylase-expressing cells (Fig. 2S and T). *Pdx1* is expressed in beta and delta cells and according to a former study also in acinar cells [15]. Thus, the Pdx1-itTA expression recapitulates the expression of the endogenous *Pdx1* gene.

**Figure 3:**
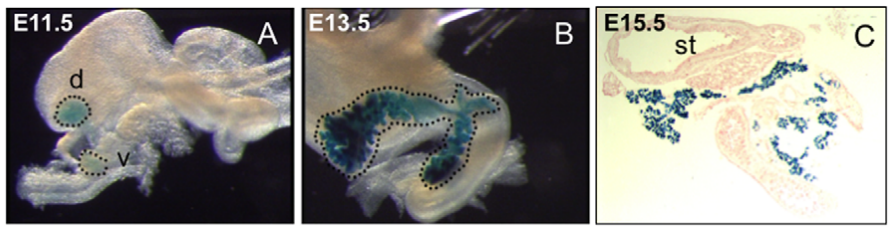
The Pdx1-itTA is active early during fetal development in pancreatic precursors. (A) LacZ expression revealed by Xgal staining in pancreatic buds at E11.5 and in the entire pancreas at E13.5 (B) and E15.5 (C) from Pdx-itTA/LacZtetOhGR fetuses. d = dorsal; v = ventral; st = stomach.

Since *Pdx1* is expressed early during pancreatic organogenesis, we analyzed the expression of Pdx1-itTA in fetuses. As early as E11.5, blue cells were detected both in the ventral and dorsal buds (Fig. 3A). These buds are separated at E11.5 but fuse later during pancreas development. At E13.5, all pancreatic cells are blue (Fig. 3B) reflecting the fact that at this stage most of the pancreatic cells are undifferentiated precursors. This staining was also observed at E15.5 (Fig. 3C).

### Generation and characterization of Ins1-itTA transgenic mice

**Figure 4:**
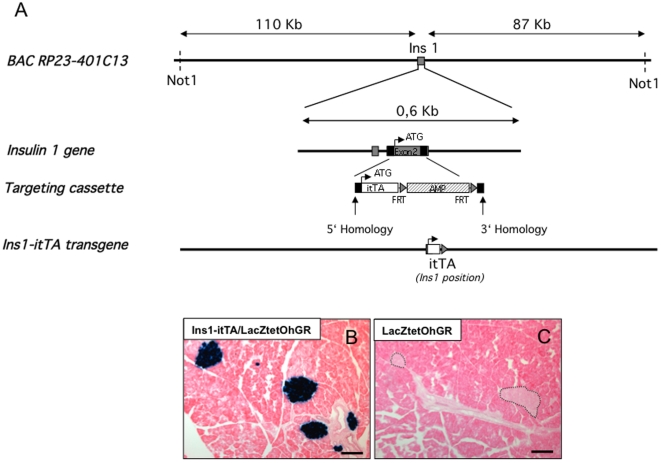
Generation and characterization of mice expressing the itTA under the control of Insulin1 regulatory elements. (A) Bacterial Artificial Chromosomes (BAC) containing around 200 kb of the genomic region of Insulin1 was obtained from BAC libraries developed by the CHORI, Oakland USA. The coding region of the gene was removed by homologous recombination in bacteria and replaced by the itTA cDNA. The construct was then injected in the pronuclei of fertilized eggs. itTA = humanized tetracycline transactivator; AMP = ampicillin resistance gene; PA = polyadenylation site; FRT = Flippase recognition target. (B) Lac Z expression revealed by Xgal staining (blue) in islets in Ins1-itTA/LacZtetOhGR mice. (C) Absence of blue staining in control mice carrying only the LacZtetOhGR transgene. Two islets are outlined. Scale bar = 50 µm.

The Insulin1 BAC clone contained 110 kb of DNA sequence upstream from the *Insulin1* gene start codon and 87 kb of DNA downstream of the polyadenylation signals sequence of the gene. This BAC clone is devoid of other known or predicted genes (Fig. 4A). The clone was modified by recombination in bacteria to introduce the *itTA* gene into the BAC at a position that ensures inactivation of the *Insulin1* gene.

The excised fragments from both Pdx1 and Insulin1 modified BACs were purified and injected into mouse C57BL/6J zygotes. We obtained and established one transgenic line harboring the Pdx1-itTA transgene and two other lines harboring the Insulin1-itTA transgene. These transgenic mice were named Tg:BAC-Ins1-itTA and will be referred in the manuscript as Ins1-itTA mice.

**Figure 5:**
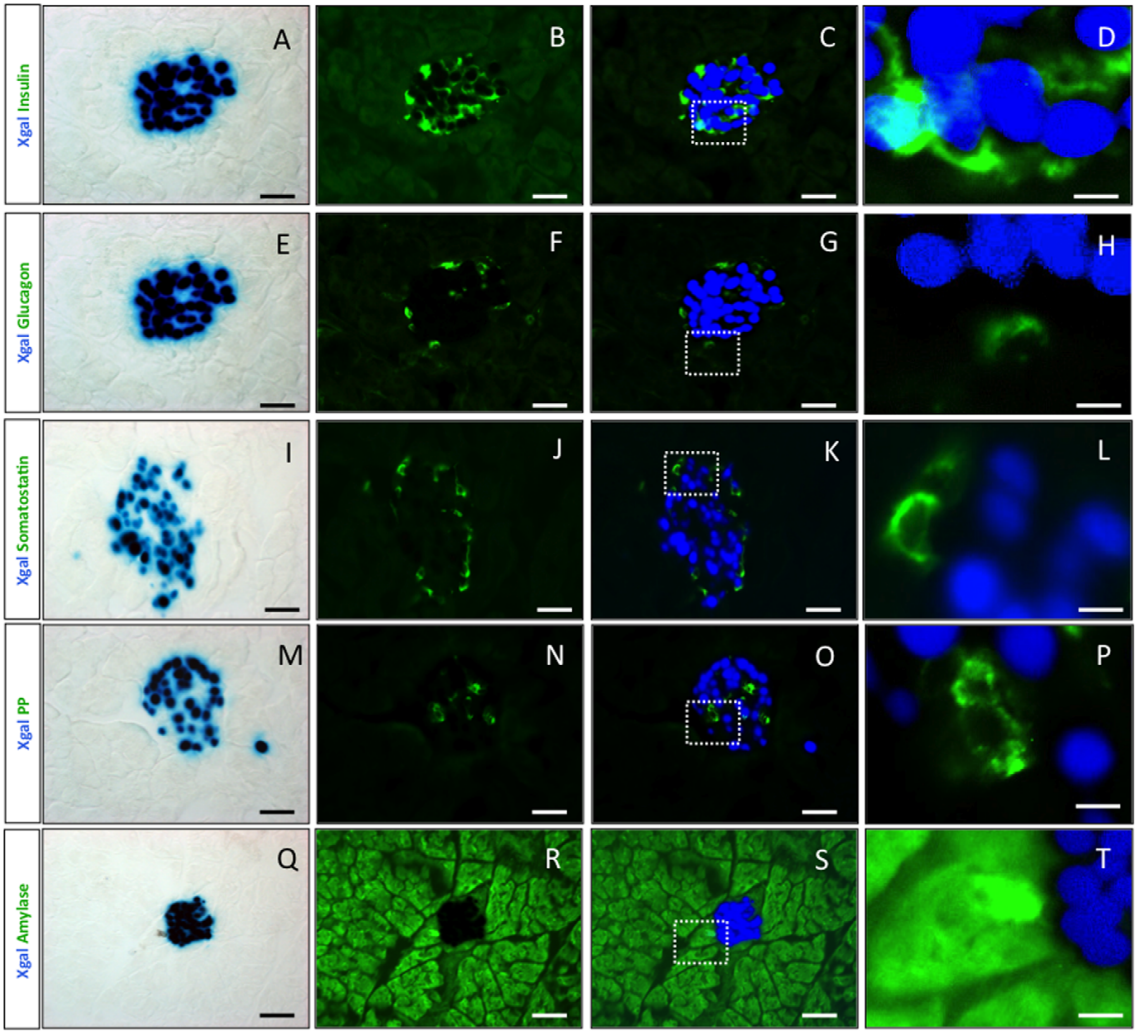
The Ins1-itTA transgene activates lacZ expression only in beta cells. (A), (E), (I), (M) and (Q) LacZ expression revealed by Xgal staining (blue) on adult pancreatic sections from a Ins1-itTA/LacZtetOhGR mouse. (B) Immunofluorescence for insulin (green), (C) merge of A and B and (D) magnified view of inset in C. (F) Immunofluorescence for glucagon (green), (G) merge of E and F and (H) magnified view of inset in G. (J) Immunofluorescence for somatostatin (green), (K) merge of I and J and (L) magnified view of inset in K. (N) Immunofluorescence for PP (green), (O) merge of M and N and P magnified view of inset in O. (R) Immunofluorescence for amylase (green), (S) merge of Q and R and (T) magnified view of inset in S. Scale bar = 50 µm except for D, H, L, P and T where scale bar = 10 µm.

**Figure 6:**
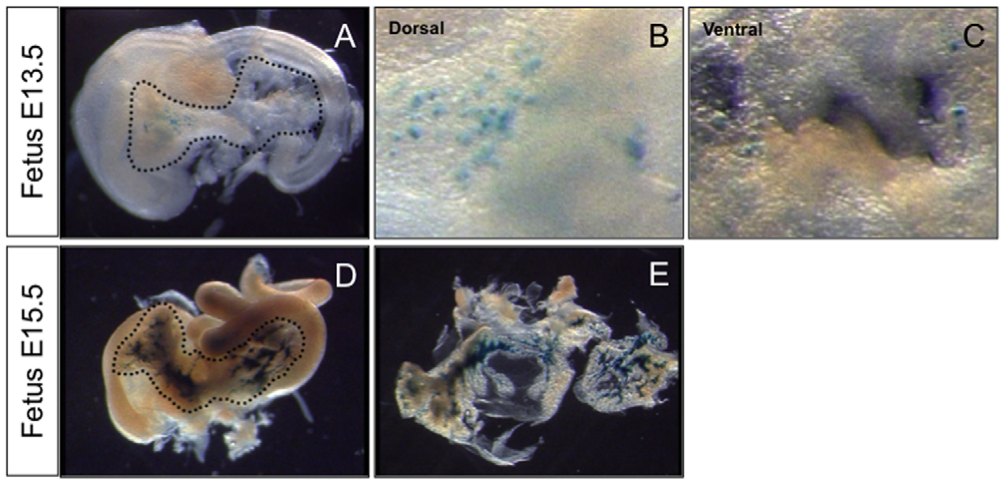
The Ins1-itTA is active in cells expressing insulin during fetal life. (A) LacZ expression in pancreatic buds at E13.5 in the dorsal (B) and ventral (C) part of the pancreas from Ins1-itTA/LacZtetOhGR fetuses. (D) LacZ expression is found at E15.5 in scattered cells. (E) A view of the dissected pancreas at E15.5.

To characterize the Ins1-itTA expression, we used the same strategy as for the Pdx1-itTA and crossed the Ins1-itTA mice with LacZtetOhGR mice. We first analyzed pancreata of mice containing both Ins-itTA and LacZtetOhGR transgenes. Xgal staining on pancreatic sections revealed a blue staining only in islets (Fig. 4B). The control mice that contain only the LacZtetOhGR transgene showed no blue staining on pancreatic sections (Fig. 4C), demonstrating that the lacZ expression is dependent upon the presence of the itTA. Analysis of other organs demonstrated no blue staining in the other organs analyzed (stomach, kidney, duodenum). To characterize the blue cells in the adult pancreas, we performed immunofluorescence on adult pancreatic sections of Ins1-itTA/LacZtetOhGR mice. We found that blue cells were positive for insulin (Fig. 5C and D) and all insulin-expressing cells were blue. In contrast, no blue cells were found in the glucagon- (Fig. 5G and H), somatostatin- (Fig. 5K and L), PP- (Fig. 5O and P) or amylase- (Fig. 5S and T) expressing cells. Thus, the expression of the Ins1-itTA is restricted to beta cells and recapitulates the expression of the endogenous *Insulin1* gene. The expression of the *Insulin1* gene starts during pancreatic organogenesis. Therefore, we analyzed the expression of Ins1-itTA in fetuses. At E11.5, no blue cells were detected in pancreatic buds from Ins1-itTA/LacZtetOhGR fetuses (data not shown). At E13.5, few scattered blue cells were found (Fig. 6A) both in the dorsal (Fig. 6B) and ventral (Fig. 6C) buds. At E15.5, clusters of blue cells were observed throughout the whole pancreas (Fig. 6D and E).

### Control of gene expression by doxycycline

**Figure 7:**
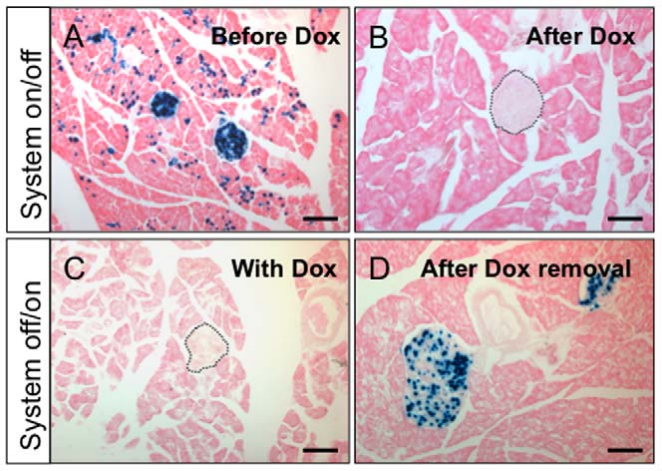
The expression of LacZ can be extinguished or induced in adult Pdx1-itTA/LacZtetOhGR mice. (A) Xgal staining on a pancreatic section of an adult Pdx1-itTA/LacZtetOhGR mouse without Dox treatment. (B) Absence of Xgal staining after 8 weeks of Dox administration (1 mg/ml) in the drinking water of an adult Pdx1-itTA/LacZtetOhGR animal. An islet is outlined. (C) Absence of Xgal staining in a 28 days-old Pdx1-itTA/LacZtetOhGR mouse treated with Dox (0.1 mg/ml) from E0. An islet is outlined. (D) Eight weeks after Dox removal, Xgal staining is observed on a pancreatic section from a Pdx1-itTA/LacZtetOhGR mouse. Note that in contrast with A, Xgal staining is observed in islet cells and not in acinar cells. Scale bar = 50 µm.

The tetracycline system can be controlled using doxycycline (Dox), an analog of tetracycline. First, we tested if lacZ expression in Pdx1-itTA/LacZtetOhGR mice could be silenced by giving Dox in the drinking water to adult mice. In the absence of Dox treatment, blue staining was observed in the pancreata of adult Pdx1-itTA/LacZtetOhGR mice (Fig. 7A). After 4 weeks of Dox treatment, no blue cells could be observed in the pancreatic sections from Pdx1-itTA/LacZtetOhGR mice (Fig. 7B), demonstrating that Dox could silence the lacZ expression.

We then tested if lacZ expression could be re-induced in the adult after silencing early in life. To do so, pregnant females carrying Pdx1-itTA/LacZtetOhGR and control LacZtetOhGR fetuses were given Dox in the drinking water from E0 (identified by a vaginal plug). Dox treatment was maintained until weaning. At weaning, we analyzed pancreata from Pdx1-itTA/LacZtetOhGR mice and did not observed any blue cells (Fig. 7C), demonstrating that Dox treatment was efficient to silence lacZ expression. After weaning, Dox treatment was stopped and mice were analyzed 8 weeks thereafter. We observed blue cells in pancreata from Pdx1-itTA/LacZtetOhGR mice (Fig. 7D) demonstrating that lacZ expression could be induced after having been silenced from E0. Interestingly, we found blue cells only in islet cells and not in exocrine cells in mice that were treated with Dox from E0 to weaning, in contrast to Pdx1-itTA/LacZtetOhGR mice that never received Dox and presented blue staining in acinar cells (Fig. 1C). This suggests that long-term transgene expression in acini depends on its transient activity during fetal pancreas development.

In separate experiments, similar controls of LacZ expression by Dox were obtained with Ins1-itTA/LacZtetOhGR mice (data not shown).

### Overexpression of the Glucocorticoid Receptor (GR) in pancreatic cells and its consequences on glucose homeostasis and beta cells

**Figure 8:**
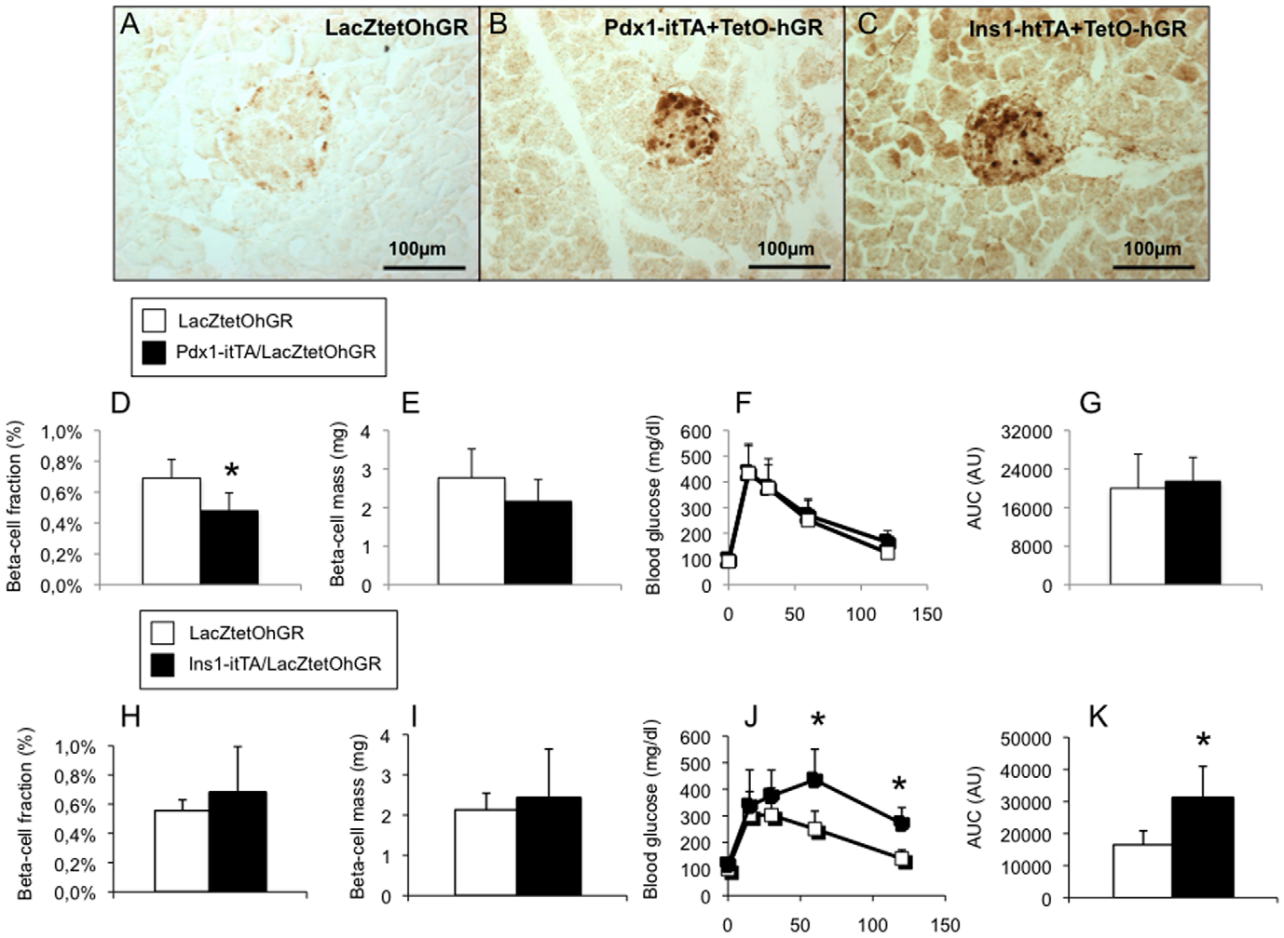
Consequences of GR overexpression on beta-cell mass and glucose homeostasis in Pdx1-itTA/LacZtetOhGR or Ins1-itTA/LacZtetOhGR mice. (A) Immunohistochemistry for the glucocorticoid receptor (GR, brown nuclear staining) on pancreatic sections from a control LacZtetOhGR, (B) a Pdx1-itTA/LacZtetOhGR mouse and (C) a Ins1-itTA/LacZtetOhGR mouse. (D) Beta-cell fraction, (E) beta-cell mass, (F) blood glucose during an intra-peritoneal glucose tolerance test and (G) corresponding Area Under the Curve (AUC) in adult Pdx1-itTA/LacZtetOhGR mice. (H) Beta-cell fraction, (I) beta-cell mass, (J) blood glucose during an intra-peritoneal glucose tolerance test and (K) corresponding Area Under the Curve (AUC) in adult Ins1-itTA/LacZtetOhGR mice. Results are expressed as means ± SD for n = 3–5 animals per group. * p<0.05 when comparing double transgenic mice (Pdx1-itTA/LacZtetOhGR or Ins1-itTA/LacZtetOhGR) to control (LacZtetOhGR) mice using a Mann-Whitney non parametric test. Scale bar = 100 µm.

After establishing that Pdx1-itTA and Ins1-itTA transgenic mice express the itTA in distinct cell populations especially during pancreatic development, we took advantage of these mice to better define the role of the glucocorticoid signaling pathway in the regulation of beta-cell mass and function. The double transgenic mice that were used to define the itTA activity express both the LacZ reporter gene and the humanized form of the Glucocorticoid Receptor (GR) under the control of the TetO. GR overexpression was confirmed by immunostaining for the GR on pancreatic sections, we observed a weak staining for a control mouse (LacZtetOhGR, Fig. 8A), and stronger stainings for a Pdx1-itTA/LacZtetOhGR (Fig. 8B) and an Ins1-itTA/LacZtetOhGR mouse (Fig. 8C) mice, confirming GR overexpression.

In Pdx1-itTA/LacZtetOhGR, GR overexpression was achieved in cells that express the itTA, more precisely the early progenitors in the fetal pancreas and beta cells. GR overexpression led to a decreased beta-cell fraction measured in adult Pdx1-itTA/LacZtetOhGR mice (Fig. 8D) with a trend for a decreased beta-cell mass (Fig. 8E) but no alteration of glucose tolerance as assessed by an intraperitoneal Glucose Tolerance Test (ipGTT, Fig. 8F and G) when compared to control LacZtetOhGR mice. Insulin secretion measured during the ipGTT 15 minutes after glucose load was comparable in the two groups (0.74±0.17 ng/ml in Pdx1-itTA/LacZtetOhGR *vs* 0.63±0.16 ng/ml in LacZtetOhGR mice).

In the second transgenic mouse model, Ins1-itTA mice, we show that GR overexpression in beta cells had no effect on beta-cell fraction (Fig. 8H) or mass (Fig. 8I). However, GR overexpression in beta cells alters their function with higher blood glucose levels during an ipGTT (Fig. 8J and K) and decreased insulin release (0.60±0.18 ng/ml in Ins1-itTA/LacZtetOhGR *vs* 0.95±0.37 ng/ml in LacZtetOhGR mice, p<0.05) 15 minutes after glucose injection. Our data thus demonstrate that GR overexpression in pancreatic cells regulates beta-cell mass or function depending on the temporal window of overexpression.

## Discussion

The use of transgenic mice is helpful to understand general biological processes and the roles of specific genes with genetic modifications such as gene deletion, gene overexpression and lineage tracing through cell labeling. More specifically, great knowledge about genes that regulate pancreatic development and beta-cell function has been gathered thanks to the use of genetic manipulation in mice (for review see [16]).

Here we present novel mice allowing conditional gene overexpression in specific pancreatic cells using genetically modified BACs. The first mouse model allows for gene overexpression early in development in pancreatic precursors, under the control of Pdx1 regulatory sequences. The second model allows for gene overexpression in beta cells only, using the *Insulin1* gene regulatory sequences.

By crossing Pdx1-itTA mice with LacZtetOhGR mice carrying a bidirectional TetO promoter that governs both LacZ reporter gene and the human GR expression, we show that itTA is expressed early in all pancreatic cells during pancreatic development and at adult age, in beta cells, delta cells and some acinar cells. This expression pattern reproduces the endogenous expression of Pdx1 (18), except for its acinar expression. Hence, by immunostaining, Pdx1 cannot be detected in acinar cells. However, a previous study has shown that the Pdx1 promoter was sufficient to drive expression of LacZ in acinar cells [15]. It is therefore possible that Pdx1 is actually expressed in acinar cells, at a level below the detection threshold of immunohistochemistry but sufficient to produce enough itTA and activate the TetO element. In agreement with this possibility, Xgal staining was indeed weaker in acinar cells than in islet cells.

In the Ins1-itTA mouse line, we show that the itTA activity is found later than in the Pdx1-itTA mouse line during pancreatic development and only in beta cells. In contrast to Pdx1-itTA mice, no expression of the itTA was detected in acinar cells. This mouse line clearly recapitulates the endogenous expression of the *Insulin1* gene.

One of the major advantages of these novel mouse lines is that they are versatile. Once generated, mice expressing the itTA in specific cells can be used to overexpress genes of interest by crossing them with mice that bear a tetracycline-regulated transgene. In the present study, we give an example of this versatility by crossing our mice with mice with a tetracycline-controlled expression of the GR, the LacZTetO-hGR mice [10]. We chose to overexpress the GR since our previous results have defined the glucocorticoids (GCs) as potent negative regulators of beta-cell development. Both in rats [4] and mice [5], we have shown that excess GCs following food restriction during late fetal development impair pancreatic beta-cell development, effects that were most probably mediated through the GR [5]. Moreover, we have shown that GR deletion specifically in beta cells (using RipCre mice) had no effect on beta-cell mass while GR deletion in pancreatic precursors (using PdxCre mice) led to a doubled beta-cell mass [3]. These results defined GCs as major inhibitors of beta-cell development. In order to further define the role of the GR on beta-cell mass and function, we overexpressed the GR in pancreatic precursors (Pdx1-itTA mouse line) or in developing and mature beta cells (Ins1-itTA mouse line). In Pdx1-itTa/LacZtetOhGR mice, we observed a decreased beta-cell mass but no effect on glucose tolerance and insulin secretion. This suggests that forced GCs signaling in pancreatic precursors influenced beta-cell differentiation and mass. On the other hand, forced GCs signaling in beta cells led to a normal beta-cell mass but impaired insulin secretion and glucose tolerance. Our present results indicate that increasing GCs signaling pathway in precursor cells leads to a long-term change in the beta-cell fraction, reinforcing the idea that a direct action of GCs in precursor cells may be the cause of the long-term changes observed after prenatal stress induced by maternal food restriction. The results obtained by overexpressing GR in mature beta cells suggest that later, GCs signaling does control beta-cell function but does not affect the beta-cell mass anymore. This demonstrates that GCs action on beta-cell mass is restricted to a window-frame, between the appearance of precursors and beta-cell differentiation. The overexpression in differentiated beta cells is in agreement with previous results obtained on transgenic mice overexpressing the GR in pancreatic beta cells and showing glucose intolerance [17].

The second advantage of the Tetracycline system we used here relies on the fact that itTA activity can be controlled by the administration of a tetracycline analog, Doxycycline (Dox). Dox treatment inhibits itTA activity and thus extinguishes the overexpression of the gene controlled by the Tetracyline-dependent promoter. Therefore, gene overexpression can be silenced or induced during specific time frames. This is of utmost importance since many genes play roles both during fetal development and in mature cells. For example, Pdx1 is a transcription factor that controls pancreas development since its deletion leads to pancreatic agenesis [18]. However, it is also expressed in mature beta cells where it controls the expression of many genes such as *Insulin* or the glucose transporter *Glut2* [19]. A good example of separate roles of genes during development or in mature cells originates from our studies on the GR. Conditional GR gene deletion in pancreatic precursors led to a doubled beta-cell mass while its inactivation in mature beta cells did not result in any phenotype, revealing separate roles of the GR gene in precursor or mature beta cells. Since we provide evidence that the itTA activity and the expression of the gene of interest that it controls can be conveniently controlled by Dox administration to the mice, further studies designed to overexpress genes during specific time frames and either in precursors or beta cells will surely reveal helpful in understanding how specific genes control beta-cell development and/or function.

One caveat of transgenic mice is the use of minimal promoters that may not reproduce the endogenous expression. For example, the rat insulin promoter (RIP) drives Cre expression both in beta cells but also in discrete brain areas [20]. More recently, thorough examination of Cre expression under the control of *Pdx1* or *Insulin* promoters has been achieved and revealed that both promoters drive the expression of Cre in brain areas [21]. The transgenes used here were constructed using BAC that contains around 200 kb of the genomic regions of *Pdx1* or *Insulin1* genes. Therefore, one can assume that such large regions contain most of the regulatory elements controlling these genes.

In conclusion, we present elegant novel mouse models that allow inducible overexpression of transgenes in selected pancreatic cell populations expressing either *Pdx1* or *Insulin1* regulatory elements. We demonstrated the versatility of these models by overexpressing the GR and revealed a dichotomy of increased GR-mediated GC signaling in the pancreas. Early action on precursors later affects beta-cell mass whereas action in mature beta cells affects function. Thus, the use of the new itTA mice presented here will help understand precise roles of genes in pancreas development and beta-cell biology.
